# *Leuconostoc mesenteroides* fermentation produces butyric acid and mediates Ffar2 to regulate blood glucose and insulin in type 1 diabetic mice

**DOI:** 10.1038/s41598-020-64916-2

**Published:** 2020-05-13

**Authors:** Supitchaya Traisaeng, Anir Batsukh, Tsung-Hsien Chuang, Deron Raymond Herr, Yu-Fen Huang, Battogtokh Chimeddorj, Chun-Ming Huang

**Affiliations:** 1 Department of Life Sciences, National Central University, Taoyuan, Taiwan; 2 Department of Biomedical Sciences and Engineering, National Central University, Taoyuan, Taiwan; 3 Immunology Research Center, National Health Research Institutes, Miaoli, Taiwan; 4 Department of Pharmacology, National University of Singapore, Singapore, Singapore; 5 Department of Biomedical Engineering and Environmental Sciences, National Tsing Hua University, Hsinchu, Taiwan; 6 Department of Microbiology and Immunology, Mongolian National University of Medical Sciences, Ulaanbaatar, Mongolia

**Keywords:** Microbiology, Bacteria, Diseases, Endocrine system and metabolic diseases

## Abstract

Type 1 diabetic patients have lower counts of butyric acid-producing bacteria in the dysbiotic gut microbiome. In this study, we demonstrate that a butyric acid-producing *Leuconostoc mesenteroides* (*L. mesenteroides*) EH-1 strain isolated from Mongolian curd cheese can reduce blood glucose and IL-6 in the type 1 diabetic mouse model. *L. mesenteroides* EH-1 fermentation yielded high concentrations of butyric acid both *in vitro* and *in vivo*. Butyric acid or *L. mesenteroides* EH-1 increased the amounts of insulin in Min6 cell culture and streptozotocin (STZ)-induced diabetic mice. Inhibition or siRNA knockdown of free fatty acid receptor 2 (Ffar2) considerably reduced the anti-diabetic effect of probiotic *L. mesenteroides* EH-1 or butyric acid by lowering the level of blood glucose. We here demonstrate that Ffar2 mediated the effects of *L. mesenteroides* EH-1 and butryic acid on regulation of blood glucose and insulin in type 1 diabetic mice.

## Introduction

Type 1 diabetes is caused by marked insulin deficiency as a result of the loss of beta cells^1–4^. Hyperglycemia in type 1 diabetes probably results from a long-term imbalance between immune-mediated beta cell damage^5^ and beta cell repair/regeneration^6^. Type 1 diabetes is characterized by the presence of hyperglycemia together with insulin resistance, oxidative stress as well as elevated production of cytokines, such as C-reactive protein, interleukin (IL)-6 and tumor necrosis factor (TNF)-α^7^.

The role of bacteria in diabetes has been presented in animal models^8^. For example, the feeding of probiotic bacteria, mostly lactic acid bacteria, to diabetes-prone rats or non-obese diabetic mice can prevent or delay diabetes^9–11^. Probiotics can reduce blood glucose through the inflammatory attenuation and prevention of pancreatic beta cell destruction *in vivo* models^12,13^. Moreover, fermentation of bacteria in human colon and mouse cecum leads to the production of short chain fatty acids (SCFAs), such as acetate, lactate, propionate and butyrate^14^. The literatures found that gut microbiome-derived SCFAs also modulate different cell types in host such as pancreatic cells^15^, immune cells^16^, adipose tissue^17^, hepatocytes^17^, muscles^17^ and neuron cells^18^. Most of these cells express SCFA receptor 2 (Ffar2) and receptor 3 (Ffar3), SCFAs are detected in the blood circulation^14,19–21^. The evidences suggest that SCFAs can manipulate such cells to regulate the health of host.

SCFAs have been proposed as therapeutic modalities against diabetes with obesity, adipose inflammation and insulin resistance^22^. Notably, butyrate supplementation increases insulin sensitivity, energy expenditure^23,24^, and the beta cell proliferation^25^. Furthermore, butyrate and butyrate-producing microbes are decreased in diabetes mellitus^26–28^. Type 1 diabetic children have a lower relative abundance of butyrate-producing bacteria^29^. In our current study, we evaluate the long term effects of the oral administration of *Leuconostoc mesenteroides* (*L. mesenteroides*), a Gram-positive bacterium referred as a *L. mesenteroides* EH-1 strain isolated from Aaruul or Mongolian curd cheese, supplement on the diabetic status of streptozotocin (STZ)-induced diabetic mice. We further evaluate the role of butyric acid, a fermentation metabolite of *L. mesenteroides* in the regulation of blood glucose in this model.

## Methods

### Bacterial culture and identification

Mongolian curd cheese was homogenized in 500 µL of sterile PBS with a grinder. Bacteria in the homogenate were cultured by plating on a tryptic soy broth (TSB) (Sigma, St. Louis, MO, USA) agar plate and incubated for 3 days at 37 °C. Sequence analysis of 16S ribosomal RNA (rRNA) genes was utilized for bacterial identification^30^. A single colony of bacteria from a TSB agar plate was isolated with a sterile toothpick and boiled at 100 °C for DNA extraction. Identification of *L. mesenteroides* EH-1 strain was validated by rRNA sequencing using the 16S rRNA 27F and 534R primers for polymerase chain reaction (PCR) (Supplementary Fig. S1)^31^. The 16S rRNA gene sequences were analyzed using the basic local alignment search tool (BLASTn, National Library of Medicine 8600 Rockville Pike, Bethesda, MD, USA). *L. mesenteroides* was cultured in TSB (Sigma) overnight at 37 °C. The cultures were diluted 1:100 and cultured to an optical density 600 nm (OD_600_) = 1.0. Bacteria were harvested by centrifugation at 5000 rpm for 10 min, washed with PBS, and suspended in PBS for further experiments.

### Glucose fermentation of *L. mesenteroides* EH-1

To induce fermentation, *L. mesenteroides* EH-1 [10^7^ colony-forming unit (CFU)/mL] was incubated in rich media [10 g/L yeast extract (Biokar Diagnostics, Beauvais, France), 5 g/L TSB, 2.5 g/L K_2_HPO_4_ and 1.5 g/L KH_2_PO_4_] in the absence or presence of 20 g/L (2%) glucose at 37 °C for 24 h. Rich media or rich media plus 20 g/L glucose without bacteria were included as a control. Phenol red [0.001% (w/v), Sigma] in rich media with 20 g/L glucose served as an indicator of fermentation, converting from red-orange to yellow when fermentation occurred. High performance liquid chromatography (HPLC) was used to quantify the level of butyric acid in cultured media.

### Min6 cell treatments

Min6 cells within 3–7 passages were cultured in Dulbecco’s modified essential medium (Gibco-BRL, Grand Island, NY, USA) with 10% (v/v) fetal bovine serum (FBS) (Irvine Scientific, Santa Ana, CA, USA), 10 mmol/L HEPES, 2 mmol/L L-glutamine, 1 mmol/L sodium pyruvate, 100 units/mL penicillin, and 100 µg/mL streptomycin. Cells were cultured for 3 days prior to analysis. After removing the media, cells were washed once with HEPES-balanced KRB (119 mmol/L NaCI, 4.74 mmol/L KCl, 2.54 mmol/L CaCl_2_, 1.19 mmol/L MgCl_2_, 1.19 mmol/L, KHaPO_4_, 25 mmol/L NaHCO_3_, 10 mmol/L HEPES, pH 7.4) containing 0.5% bovine serum albumin (BSA) without glucose. Min6 cells were preincubated for 0.5 h in 4-(2-hydroxyethyl)-1-piperazineethanesulfonic acid (HEPES)-balanced Krebs ringer buffer (KRB). After washing twice with HEPES-balanced KRB, Min6 cells were incubated for 24 h in HEPES-balanced KRB supplemented with 0.5% BSA and 100 µmol/L glucose or 100 µmol/L butyric acid or propionic acid. The media were then collected and assayed by a mouse insulin ELISA kit. For small interfering RNA (siRNA)-mediated knockdown of Ffar2, siRNA against Ffar2 was purchased from GenePhama, Shanghai, China. Min6 cells (10^5^ cells/mL) within 5–7 passages were cultured for 3 days and then reversely transfected with 10 µmol/L of Ffar2 (sense strand: 5′-GCUGUUGUGACGCUUCUUATT-3′ and anti-sense strand: 5′-UAAGAAGCGUCACAACAGCTT-3′) or scramble (sense strand: 5′-UUCUCCGAACGUGUCACGUTT-3′ and anti-sense strand: 5′ACGUGACACGUUCGGAGAATT-3′) siRNAs using Lipofectamine 2000, and the media were changed 6 h thereafter. A second transfection with siRNA followed on the second day, and the experiment was performed 48 h after the second transfection as described above. RNA was extracted for quantification of Ffar2 expression by real-time PCR (RT-PCR).

### Streptozotocin (STZ)-induced type 1 diabetic mice

The Institute Cancer Research (ICR) mice (8–12 week-old males; National Laboratory Animal Center, Taiwan) were housed at 25 °C with a 12:12 h light-dark cycle, fed a normal chow diet, and given water ad libitum. Mice (n = 4/group) were acclimatized for 5–7 days before the experiment. To induce a rapid ablation of the beta cells and hyperglycemia^32^ and avoid the interruption of STZ with *L. mesenteroides* EH-1 or butyric acid, mice were injected with a single dose, instead of low multiple doses^33^, of STZ. Diabetes was induced following an 8-h fast using a single intraperitoneal (IP) injection of STZ (200 mg/kg body weight) (Sigma)^32^, which was dissolved in acidified citrate buffer (0.1 mol/L, pH 4.5). Two days later, after a 4-h-fast, the level of blood glucose from the tail blood was measured using a glucometer (Advantage Glucometer, Roche, Mannheim, Germany). Injection of STZ for 2 days induced a weight lost (32.8 ± 0.9 vs 29.6 ± 1.2 mg; with vs without STZ). Mice with glucose levels of 200 mg/dL or greater were recruited into the diabetic group^34^. This research was carried out in strict accordance with an approved Institutional Animal Care and Use Committee (IACUC) protocol at National Central University (NCU), Taiwan (NCU-106-015, 19 December 2017). Fasting retro-orbital sinus blood was collected in heparinized tubes and then centrifuged at 3,000 rpm for 15 min. Plasma was stored at −80 °C until use. A mouse IL-6 ELISA kit (R&D systems, Minneapolis, MN, USA) was used to detect the levels of IL-6. The level of insulin was detected using a mouse insulin ELISA kit (Mercodia, Uppsala, Sweden) (Supplementary Fig. S4).

### Diabetic mice treated with butyric acid

Butyric acid at 4 mmol/L in water (5 mL/kg body weight) was administrated to diabetic mice via IP injection. Mice in control groups received water alone. Blood glucose was detected every day. Seven days after butyric acid injection, fasting blood was collected for detection of insulin and IL-6 as described above. For Ffar2 inhibition, a Ffar2 antagonist (GLPG-0974, Tocris Bioscience, Bristol, UK) was dissolved in dimethylsulfoxide (DMSO) to make a stock solution. Diabetic mice were injected with butyric acid (5 mL/kg body weight) taken from a stock solution of 4 mmol/L. GLPG-0974 (1 mg/kg body weight) was diluted in saline then was given at 1 mL/kg body weight^35^ by gastric gavage just before butyric acid injection. DMSO (0.1% in saline) was used as a vehicle control. Twenty-four h after butyric acid injection, blood was collected for the detection of glucose, insulin and IL-6 levels.

### Feeding mice with *L. mesenteroides* EH-1

ICR mice were fed with live or heat (100 °C)-killed *L. mesenteroides* EH-1 (8 × 10^9^ CFU/50 µL) once a day for 2 days. Water (50 µL) was given as control. Two days after the *L. mesenteroides* EH-1 feeding, cecum was homogenized in water (50 mg/500 µL), followed by a vortex mixing step, and stored at −80 °C until the detection of butyric acid by HPLC. Two weeks after daily feeding diabetic mice with *L. mesenteroides* EH-1, blood glucose was detected once a week, fasting blood was collected for the detection of insulin and IL-6 levels. Mice were sacrificed in a CO_2_ chamber and the pancreases were collected for immunohistochemical analysis. For Ffar2 inhibition, diabetic mice were fed with *L. mesenteroides* EH-1 (8 × 10^9^ CFU/50 µL) once a day for 2 weeks. GLPG-0974 was given at 1 mL/kg body weight by gastric gavage just before *L. mesenteroides* EH-1 administration and weekly. A vehicle was 0.1% DMSO in saline. Blood glucose was detected once a week. Two weeks after treatment, blood was collected for detection of insulin and IL-6 levels.

### Statistical analysis

To determine significance between groups, comparisons were made using the two-tailed Student’s t-test. Data are presented as mean values ± standard deviation (SD). The mean values ± SD for all figures with bar charts were shown in Supplementary Table 1. Statistical analyses were performed using GraphPad Prism 5 software. Unpaired Student’s t-test was used to compare two groups. When appropriate, ANOVA was used and post hoc analysis was performed with Tukey’s test to compare more than two groups. A *p* value < 0.05 was regarded as statistically significant.

## Results

### Fermentation properties of *L. mesenteroides* EH-1

**Figure 1:**
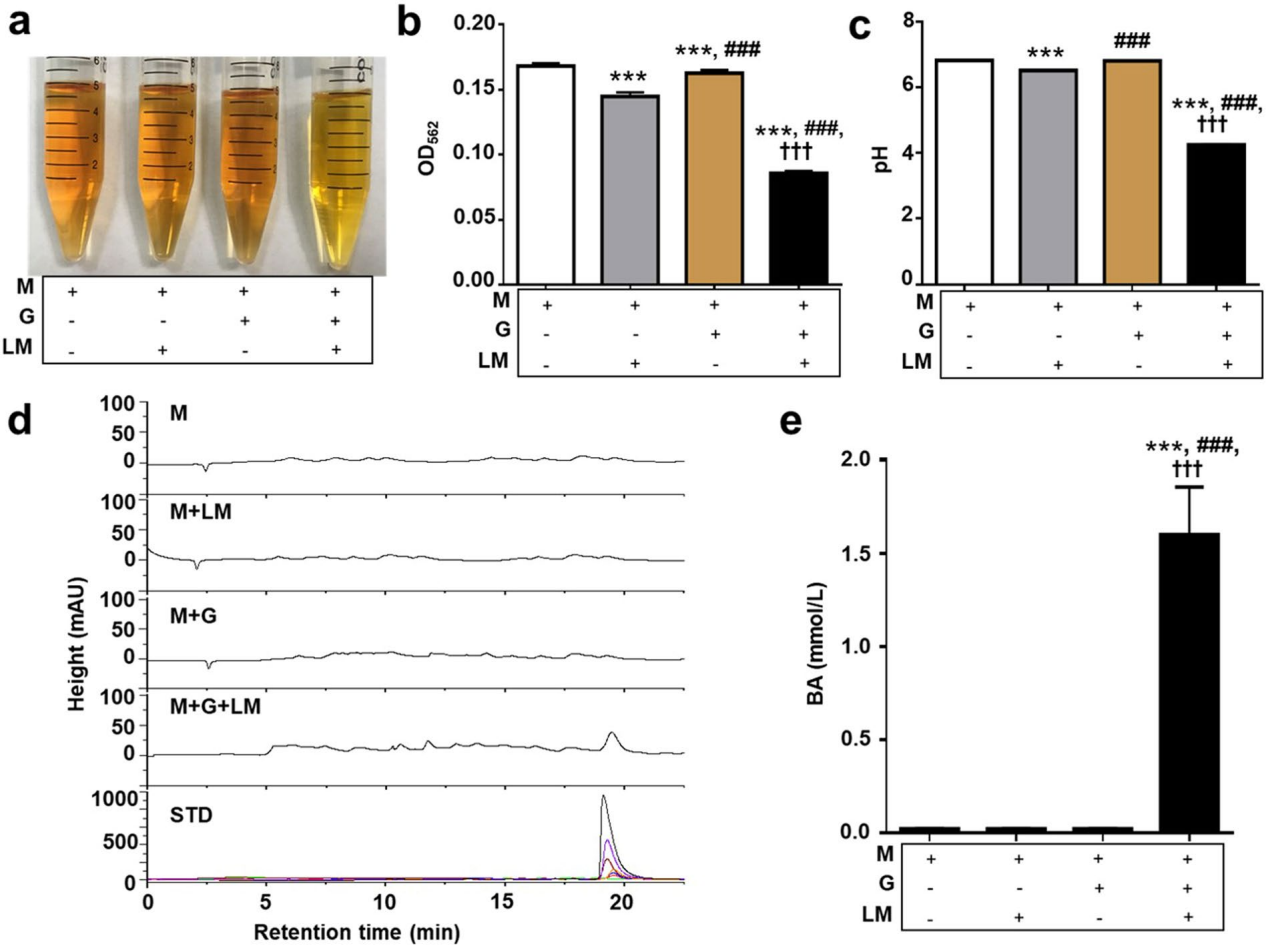
Glucose fermentation of *L. mesenteroides* EH-1. (**a**) *L. mesenteroides* EH-1 (LM) was incubated in rich media (M) with/without glucose (G) for 24 h. Rich media alone and rich media plus glucose without *L. mesenteroides* EH-1 were included as controls. Fermentation was detected by (**b**) OD_565_ and (**c**) pH value. (**d**) Butyric acid was measured by HPLC and (**e**) the concentrations of butyric acid were calculated from the height of butyric acid standard (STD) peaks. Data are the mean ± SD from 3 independent experiments. ***p < 0.001 vs M, ^**###**^*p* < 0.001 vs M + LM, and ^**†††**^*p* < 0.001 vs M + G.

A single colony was isolated from a TSB agar plate spread with Mongolian curd cheese and evaluated by 16S rRNA sequencing. The 16S rRNA gene (Supplementary Fig. S1) of this colony shares 99% identity to that of *L. mesenteroides* ATCC 8293. This isolated strain was here named as *L. mesenteroides* EH-1. *L. mesenteroides* is a lactic acid bacterium that is currently used as a starter for kimchi and kefir^36^. *L. mesenteroides* EH-1 grew well at the temperatures of 25 °C and 37 °C, but not 4 °C (Supplementary Fig. S2). The growth of *L. mesenteroides* EH-1 was unaffected by acidification of the media, as growth curves were similar from pH 3 to pH 7 (Supplementary Fig. S3). These results indicate that the strain of *L. mesenteroides* EH-1 isolated from Mongolian curd cheese is stable at room temperature and tolerant of low pH. To examine the fermentative capabilities, *L. mesenteroides* EH-1 was cultured in rich media in the presence of 2% glucose for 24 h. Rich media with glucose alone or *L. mesenteroides* EH-1 alone served as controls. The media in the culture of *L. mesenteroides* EH-1 with glucose turned yellow after incubation for 24 h, while the media in the other three conditions maintained their original colors (Fig. 1a). As shown in Fig. 1b,c, the OD_562_ and pH values of media with *L. mesenteroides* EH-1 plus glucose demonstrated significant decreases compared to controls, indicating that *L. mesenteroides* EH-1 has a capability of fermenting glucose. HPLC analysis was conducted to quantify the level of butyric acid in fermentation media of *L. mesenteroides* EH-1. Butyric acid is detectable in media from glucose fermentation of *L. mesenteroides* EH-1, but not media from controls (Fig. 1d). The different concentrations of butyric acid (0–100 mmol/L) were subjected to HPLC for establishment of a quantitative standard curve. As shown in Fig. 1e, glucose fermentation of *L. mesenteroides* EH-1 for 24 h yielded approximately 1.6 mmol/L of butyric acid.

### Effects of butyric acid on insulin secretion from Min6 cells

**Figure 2:**
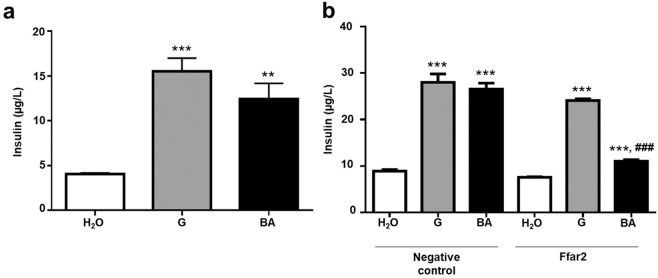
Effects of butyric acid on insulin secretion from Min6 cells. (**a**) Levels of insulin secretion from Min6 cells after treatments with water (H_2_O), 100 µmol/L glucose (G), and 100 µmol/L butyric acid (BA) for 24 h were measured by ELISA. (**b**) Min6 cells pre-treated with Ffar2 or negative control siRNAs before incubation with water, glucose, and butyric acid for 24 h. The level (µg/L) of insulin was detected using a mouse insulin ELISA kit. Data are the mean ± SD from 3 independent experiments. **p < 0.01; ***p < 0.001 vs water treatment and ^#^*p* < 0.05; ^###^*p* < 0.001 vs glucose treatment.

To investigate the effect of butyric acid on insulin secretion, Min6 cells were treated with butyric acid (100 mmol/L) for 24 h. Treatment of glucose or water served as positive and negative controls, respectively. Results in Fig. 2a showed that treatment of cells with butyric acid, like glucose, markedly elevated insulin levels in culture media of Min6 cells. To determine whether Ffar2 mediated the regulation of butyric acid on insulin secretion, cells were pre-treated with Ffar2 or scrambled siRNA before addition of water alone, glucose alone, or butyric acid plus glucose for 24 h. The Ffar2 siRNA induced a 74.46 ± 2.27% knockdown of Ffar2 gene (Supplementary Fig. S5). As shown in Fig. 2b, the knockdown of Ffar2 with its specific siRNA, but not scrambled siRNA, considerably blocked the effect of butyric acid on induction of insulin secretion. On the other hand, the Ffar2 knockdown had no influence on glucose-induced insulin secretion from Min6 cells. The results suggest that Ffar2 is essential for the action of butyric acid at induction of insulin secretion from Min6 cells.

### Involvement of Ffar2 in the effect of butyric acid on the levels of blood glucose, and insulin in type 1 diabetic mice

**Figure 3:**
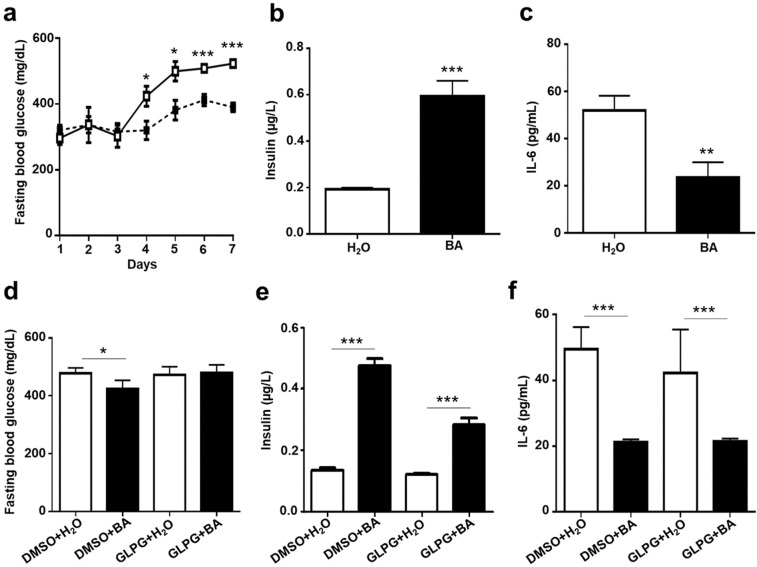
Effects of butyric acid on blood glucose, insulin, and IL-6 levels in diabetic mice. Diabetic mice were injected with water (H_2_O; open square/bar) or butyric acid (BA; solid square/bar) once a day for a week. (**a**) Fasting blood glucose was quantified daily. After 7 days, fasting blood was collected for (**b**) insulin and (**c**) IL-6 detection by ELISA. Diabetic mice were given a single dose of GLPG-0974, an Ffar2 antagonist, with water or butyric acid for 24 h via IP injections. 0.1% DMSO in saline was used as vehicle control. Fasting blood was collected for detection of (**d**) glucose, (**e**) insulin and (**f**) IL-6 detection two weeks after treatments. Data are the mean ± SD from 3 independent experiments with 4 mice per group. *p < 0.05; **p < 0.01; ***p < 0.001 vs water treatment.

To establish a type 1 diabetic mouse model, STZ was administered to ICR mice via IP injection. Compared to control mice, injection of STZ for two days led to higher levels of glucose and IL-6 in the blood. STZ-induced diabetic mice were injected intraperitoneally with butyric acid or water once a day for a week. Injection of butyric acid resulted in a remarkable decrease in fasting blood glucose (Fig. 3a) as well as IL-6 (Fig. 3c). Furthermore, the amount of insulin in the plasma of butyric acid-injected mice was higher than that of water-injected mice (Fig. 3b). To further confirm the essential role of Ffar2 in mediating the action of butyric acid *in vivo*, the STZ-induced diabetic mice were administered intragastrically with GLPG-0974, an Ffar2 antagonist, or DMSO control before injection of butyric acid or water. Administration of GLPG-0974, but not DMSO, counteracted the effect of butyric acid on the down-regulation of glucose and the up-regulation of insulin (Fig. 3d,e), although no effect on butyric acid-induced IL-6 reduction was observed (Fig. 3f). These results indicate that butyric acid may regulate the levels of glucose and insulin in blood of STZ-induced diabetic mice via binding to Ffar2.

### Production of butyric acid by *L. mesenteroides* EH-1 *in vivo*

**Figure 4:**
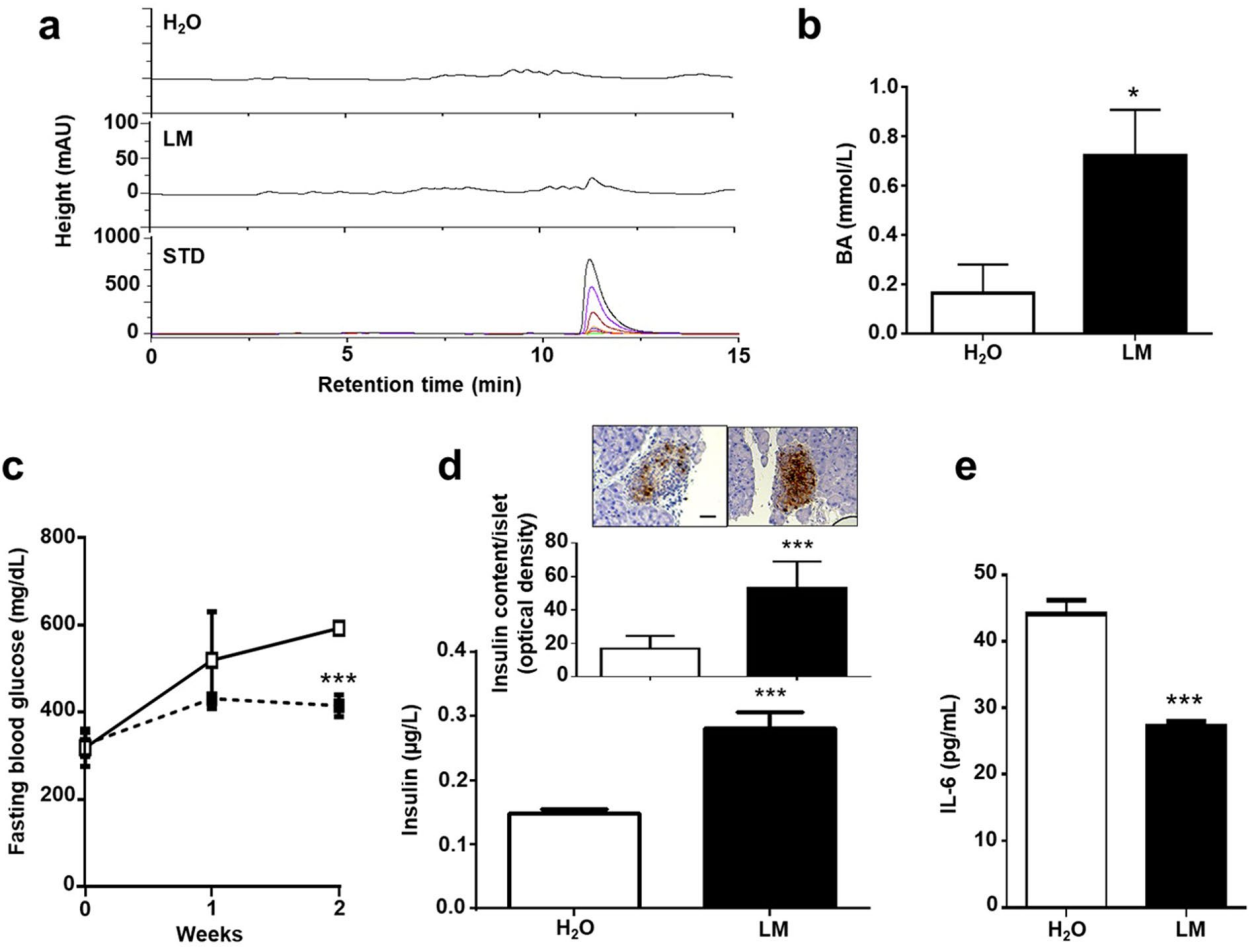
Effects of feeding live *L. mesenteroides* EH-1 on blood glucose, insulin, and IL-6 levels. (**a**) ICR mice were fed *L. mesenteroides* EH-1 (LM; solid square/bar) or water (H_2_O; open square/bar) alone once a day for 2 days and then the cecum was collected for butyric acid detection by HPLC. (**b**) The concentration of butyric acid was calculated from the height of butyric acid standard peaks. The levels of (**c**) glucose, (**d**) insulin and (**e**) IL-6 in the fasting blood were detected after feeding diabetic mice with water or *L. mesenteroides* EH-1 once a day for 2 weeks. (**d**) The tissue sections (D; inserts) of mouse pancreas were used to detect islet insulin content using immunohistochemistry (scale bars, 50 μm). Data are the mean ± SD from 3 separate experiments with 4 mice per group. *p < 0.05; ***p < 0.001.

**Figure 5:**
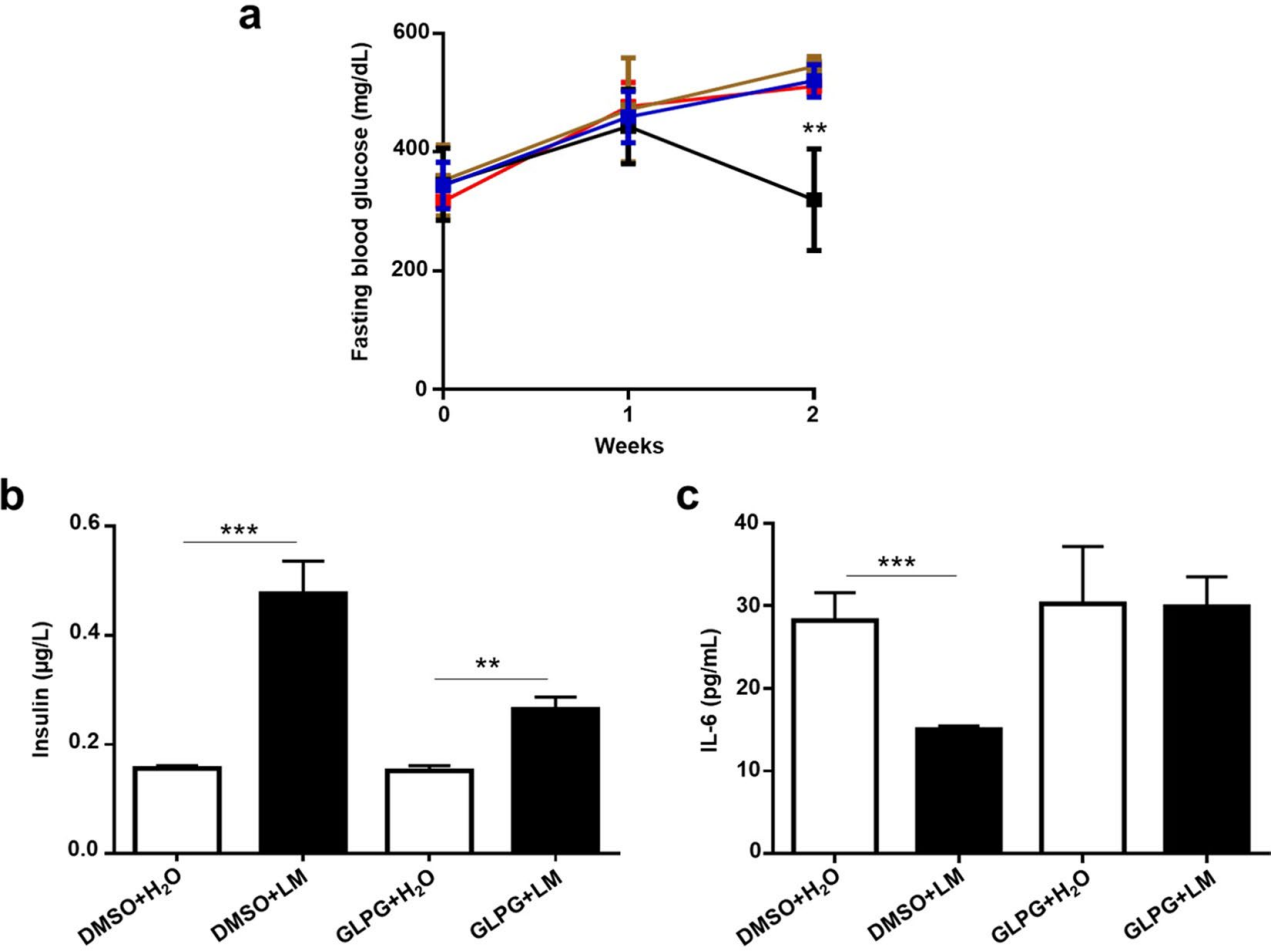
The levels of glucose, insulin and IL-6 in blood of Ffar2-inhibited diabetic mice. STZ-induced diabetic mice were given GLPG-0974 (GLPG) once a week. Right after GLPG-0974 treatment, mice were fed with water (H_2_O) or *L. mesenteroides* EH-1 (LM) once a day for 2 weeks. DMSO (0.1% in saline) was used as a vehicle control. The (**a**) glucose (blue, DMSO + H_2_O; black, DMSO + *L. mesenteroides* EH-1; brown, GLPG + H_2_O; red, GLPG + *L. mesenteroides* EH-1), (**b**) insulin and (**c**) IL-6 in fasting blood were detected. Data are the mean ± SD from 3 independent experiments with 4 mice per group. **p < 0.01; ***p < 0.001 vs water treatment.

To explore whether butyric acid can be produced *in vivo* by *L. mesenteroides* EH-1, ICR mice were fed with *L. mesenteroides* EH-1 or water once a day for 2 days. Butyric acid in cecum homogenates was measured by HPLC. Butyric acid is detectable in the cecum of *L. mesenteroides* EH-1-fed mice, but not in that of control mice fed with water (Fig. 4a,b). Approximately 0.7 mmol/L butyric acid was detected in the cecum, suggesting that *L. mesenteroides* EH-1 can produce butyric acid at a high concentration in a cecum microenvironment. To assess whether butyric acid-producing *L. mesenteroides* EH-1 can lower the blood glucose in diabetes, STZ-induced diabetic mice were fed with *L. mesenteroides* EH-1 or water. Feeding mice with *L. mesenteroides* EH-1 once a day for 2 weeks substantially reduced the levels of glucose (Fig. 4c) and IL-6 in the blood (Fig. 4e) and increased the amounts of insulin (Fig. 4d) in the blood and pancreas (Fig. 4d, inserted panels). These results demonstrate the probiotic activity of *L. mesenteroides* EH-1 in regulating the levels of glucose and IL-6 in STZ-induced type 1 diabetic mice. To validate the contribution of Ffar2 to the effect of butyric acid-producing *L. mesenteroides* EH-1 on lowering blood glucose, GLPG-0974 was given to STZ-induced diabetic mice to antagonize the Ffar2 before feeding mice with *L. mesenteroides* EH-1. Compared to mice treated with DMSO control, mice given GLPG-0974 displayed no difference in glucose (Fig. 5a), IL-6 (Fig. 5c), as well as insulin (Fig. 5c) in blood. Taken together, the data in Figs. 3 and 5 strongly suggest that Ffar2 mediates the signaling of butyric acid produced by *L. mesenteroides* EH-1 to diminish the elevated glucose in STZ-induced diabetic mice.

## Discussion

Probiotic *L. mesenteroides* is currently used for food fermentation. An addition of *L. mesenteroides* to cabbage fermentation ensured that texture and flavor quality were retained, while providing a 50% reduction in sodium chloride^37^. In addition, this probiotic bacterium has demonstrated a number of beneficial effects, including inhibition of three fish pathogens^38^, suppression of low‐pathogenic avian influenza (H9N2) virus in chickens^39^, and reduction of *Streptococcus thermophilus* induced IL-12 and interferon (IFN)-γ production in human peripheral blood mononuclear cells^40^.

Previous studies have identified potential antidiabetic effects of other commensal bacteria. Treatment with *Lactobacillus casei* CCFM419 improved impaired pancreatic function and attenuated type 2 diabetes^41^ in a mouse model. Carrot juice fermentation of *Lactobacillus plantarum* NCU116 reduced pancreatic injuries^42^. Oral administration *Lactobacillus rhamnosus* CCFM0528 improved glucose intolerance by protecting islet cells^43^. Our current study demonstrated for the first time that *L. mesenteroides* EH-1 exerts a probiotic activity that lowers blood glucose in the STZ-induced type 1 diabetic mouse model. To validate the essential role of *L. mesenteroides* EH-1 in regulation of glucose and insulin levels in diabetic mice, we fed STZ-induced type 1 diabetic mice with heat-killed *L. mesenteroides* EH-1. As shown in Supplementary Fig. S7, the killed *L. mesenteroides* EH-1 bacteria lost their capacity to lower glucose and increase insulin, indicating that *L. mesenteroides* EH-1, not endogenous gut microbes indirectly affected by probiotic *L. mesenteroides* EH-1, exerted the anti-diabetic properties. Future works will use germ-free mice^44^ or mice with the gut microbiome depleted by antibiotics^45^ to study the effects of probiotic bacteria and endogenous gut microbes on the regulation of blood glucose and insulin.

Ffar2 is highly expressed on pancreatic islets^46–48^ and up-regulated in islets during pregnancy^49^. The potency rank order of SCFAs for Ffar2 is acetate (C2) ∼ propionate (C3) > butyrate (C4) > valerate (C5) > formate^50^. Moreover, Ffar2 is a novel effector of glucose homeostasis in part due to its direct effect on insulin secretion and beta cell proliferation^51,52^. In agreement with previous studies, our data revealed that treatment of Min6 cells with 100 mmol/L butyric acid for 24 h provoked the insulin secretion (Fig. 2a). By conducting the oral glucose tolerance test (OGTT) in diabetic mice (Supplementary Fig. 8), we found that injection of butyric acid, but not water, before oral administration of glucose promoted the metabolism of plasma glucose and elevated the level of insulin in the blood. The high level of insulin in the blood could be due to an increase in insulin secretion from pancreas or a decrease in insulin clearance in the liver^53^. It has been reported that sodium butyrate supplementary diet down regulated insulin receptor (IR), and IR substrate 1 (IRS-1) expression^54^ involved in insulin signaling in the mouse liver^55^. Application of butyrate also associated with decreased insulin receptor beta subunit (IR-bata) expression of in hepatic tissue^56^. Thus, administration of butyric acid into mice may enhance the insulin secretion and prolong the insulin clearance in mice.

Butyric acid is one of Ffar2’s known agonists. Previous studies have shown that Ffar2 agonism can trigger an increase in intracellular inositol triphosphate and Ca^2+^ levels, and potentiate insulin secretion^52^. In addition, sodium butyrate treatment improved glucose homeostasis and reduced beta cell apoptosis in diabetic rats^25^. Knockdown of Ffar2 significantly reduced butyric acid-induced insulin secretion from Min6 cells, clearly illustrating that Ffar2 signaling is an important effector of insulin secretion induced by butyric acid. Previous studies have demonstrated a strong reduction in plasma glucose by feeding with ketogenic diets. Both propionic acid and butyric acid^57^ are ketogenic substrates and can bind to Ffar2^50^. The effects of propionic acid and butyric acid on insulin secretion from Min6 cells were compared side-by-side. Propionic acid induced detectable amounts of insulin secreted from Min6 cells although it is less effective than butyric acid (Supplementary Fig. S6). Knockdown of Ffar2 considerably lowered propionic acid-induced insulin secretion from Min6 cells. Butyric acid, but not propionic acid, is a potent inhibitor of histone deacetylases (HDAC)^58^. Previous studies demonstrated that butyrate can improve insulin sensitivity via HDAC inhibition^24^. Butyrate as a dietary supplement can prevent high fat diet-induced insulin resistance in mice by promotion of energy expenditure and induction of mitochondria function^24^. Fasting insulin was significantly lower in the butyrate-treated high-fat diet mice. The signal of phosphorylation of IRS-1 in the skeletal muscle was increased in butyrate-treated mice, suggesting a molecular mechanism of insulin sensitization. Our data revealed that butyric acid can mediate Ffar2 to increase blood insulin in STZ-induced type 1 diabetic mice. Thus, it is worth investigating whether butyric acid controls the activity of Ffar2 or HDAC to regulate the insulin secretion in different types of diabetes.

Loss of Ffar2 in mice increases the risk of diabetic status, since Ffar2 knockout (KO) mice exhibit fasting hyperglycemia, reduced insulin levels, and glucose intolerance, despite exhibiting normal insulin sensitivity^52^. Exposing Ffar2 KO mice to a high fat diet resulted in a decrease in both islet number and size, leading to reduced beta cell mass and total pancreatic insulin content. A butyrate-enriched diet could partially protect Ffar2 KO mice in a non-obese diabetic background from type 1 diabetic islet inflammation^59^. As shown in Fig. 3, a single dose of GLPG-0974, a Ffar2 antagonist, suppressed butyric acid-induced increase of fasting blood insulin and decreased fasting blood glucose, confirming the indispensable role of Ffar2 in the action of butyric acid *in vivo*. Furthermore, Ffar2 activation on intestinal enteroendocrine cells induces the production of glucagon-like peptide (GLP)-1 which is anorexigenic and stimulates insulin secretion^60^. Future work is required to determine if GLP-1 mediates the Ffar2-butyric acid induced insulin secretion in the STZ-induced type 1 diabetic mouse model.

STZ specifically damages pancreatic islet beta cells. The damaged beta cells passively relate to high mobility group box 1 (HMGB1) release^61^. In parallel, the inflammatory cells infiltrated pancreatic islets, such as macrophages, dendritic cells and T cells. The differentiation of Naïve CD4+ T cells into effector T helper (Th)1 and/or Th17 cells based on current cytokine microenvironment. In addition, the released HMGB1 targets macrophage or dendritic cells via the corresponding surface receptor(s), which induces a cascade signal that activates the nuclear factor κB (NF-κB) pathway^62^. This results in the activation of Th17 cells, therefore leading to the production of proinflammatory cytokines IL-1β, TNF-α, and IL-6^63^. It has been reported that IL-6 induces beta cell apoptosis via signal transducer and activator of transcription (STAT)-3-mediated the production of nitric oxide^64^. The dysregulation of proinflammatory cytokines and Th1/Th2/Th17 further accelerate an inflammation of islets and destruction of beta cells, and leads to type 1 diabetes. Sodium butyrate can heal the balance of Th1/Th2 and block Th17 cells^65^.

In this study, we found that the acute blocking of Ffar2 by GLPG-0974 at a single dose for 24 h showed a minor effect of Ffar2-butyric acid signaling on IL-6 reduction (Fig. 3f), whereas the long term blocking of Ffar2 using multiple doses of GLPG-0974 for two weeks resulted in a significant the suppression effect on the *L. mesenteroides* EH-1-reduced blood IL-6 level in type 1 diabetic mice (Fig. 5c). Although other Ffar2 antagonists from azetidine derivatives^66^ at different doses can be used to completely block Ffar2 *in vivo*, it has been documented that sodium butyrate, as a direct HMGB1 antagonist, could down-regulate the expression of HMGB1 and mediate the balance of Th1/Th2/Th17 paradigm, thus attenuating type 1 diabetes. Thus, it is possible that butyric acid reduced IL-6 production by directly down-regulating HMGB1 expression and bypassing Ffar2.

STZ does not influence the function of pancreatic beta cells of humans when used in the treatment of islet-cell carcinomas^67^. Insulin can be detected in STZ-injected mice (Fig. 4d), indicating that STZ injection results in incomplete damage to the pancreas in mice. It has been shown that Ffar2 directly mediates both the stimulatory effects of sodium acetate and propionate on insulin secretion and their protection against islet apoptosis^68^. In addition to butyric acid, other SCFAs in the fermentation media of *L. mesenteroides* EH-1 will be measured in the future. One of limitations of using SCFAs as therapeutics includes their typically short *in vivo* half-life, with clearance from plasma occurring within a few hours^69^. A high concentration (about 0.7 mmol/L) of butyric acid was detected in the cecum of mice fed with *L. mesenteroides* EH-1 (Fig. 4a), demonstrating that *L. mesenteroides* EH-1 is a potent strain for producing butyric acid. Data in literature demonstrated that high-fat-fed mice treated with butyrate showed enhancement of the insulin secretion, which was related to a substantial reduction in lipid accumulation within the pancreas^70^. Furthermore, butyrate can prevent type 1 diabetes in non-obese diabetic (NOD) mice^59^ Results from previous studies above supported that butyric acid has a beneficial effect on prevention of type 1 diabetes. Future works will test the anti-diabetic activity of probiotic *L. mesenteroides* EH-1 using NOD mice or high-fat diet-induced type 1 diabetes.

In summary, our study characterized a new probiotic bacterial strain, *L. mesenteroides* EH-1, which was originally isolated from Mongolian curd cheese. *L. mesenteroides* EH-1 produces high concentrations of butyric acid which can activate Ffar2 to raise insulin levels but mitigate glucose amounts in the blood of type 1 diabetic mice.

## Supplementary Information

Supplementary Information.

## References

[1] Warren S, Root HF (1925). The pathology of diabetes, with special reference to pancreatic regeneration. Am. J. Pathol..

[2] Gepts W (1965). Pathologic anatomy of the pancreas in juvenile diabetes mellitus. Diabetes.

[3] Junker K, Egeberg J, Kromann H, Nerup J (1977). An autopsy study of the islets of Langerhans in acute‐onset juvenile diabetes mellitus. Acta Path. Microbiol. Scand. Sect. A.

[4] Pipeleers D, Ling Z (1992). Pancreatic beta cells in insulin‐dependent diabetes. Diabetes Metab. Rev..

[5] Kukreja A, Maclaren NK (1999). Autoimmunity and diabetes. J. Clin. Endocrinol. Metab..

[6] Eizirik DL, Sandler S, Palmer JP (1993). Repair of pancreatic β-cells: a relevant phenomenon in early IDDM?. Diabetes.

[7] Goldberg RB (2009). Cytokine and cytokine-like inflammation markers, endothelial dysfunction, and imbalanced coagulation in development of diabetes and its complications. J. Clin. Endocrinol. Metab..

[8] Brugman S (2006). Antibiotic treatment partially protects against type 1 diabetes in the Bio-Breeding diabetes-prone rat. Is the gut flora involved in the development of type 1 diabetes?. Diabetologia.

[9] Matsuzaki T (1997). Prevention of onset in an insulin‐dependent diabetes mellitus model, NOD mice, by oral feeding of Lactobacillus casei. Apmis.

[10] Calcinaro F (2005). Oral probiotic administration induces interleukin-10 production and prevents spontaneous autoimmune diabetes in the non-obese diabetic mouse. Diabetologia.

[11] Yadav H, Jain S, Sinha P (2007). Antidiabetic effect of probiotic dahi containing Lactobacillus acidophilus and Lactobacillus casei in high fructose fed rats. Nutrition.

[12] Al-Salami H (2008). Probiotic treatment reduces blood glucose levels and increases systemic absorption of gliclazide in diabetic rats. Eer. J. Drug Metab. Ph..

[13] Zhang Q, Wu Y, Fei X (2016). Effect of probiotics on glucose metabolism in patients with type 2 diabetes mellitus: a meta-analysis of randomized controlled trials. Medicina.

[14] Morrison DJ, Preston T (2016). Formation of short chain fatty acids by the gut microbiota and their impact on human metabolism. Gut microbes.

[15] Priyadarshini M, Wicksteed B, Schiltz GE, Gilchrist A, Layden BT (2016). SCFA receptors in pancreatic β cells: novel diabetes targets?. Trends Endocrinol..

[16] Bhutia YD, Ganapathy V (2015). Short, but smart: SCFAs train T cells in the gut to fight autoimmunity in the brain. Immunity.

[17] den Besten G (2013). The role of short-chain fatty acids in the interplay between diet, gut microbiota, and host energy metabolism. J. Lipid Res..

[18] Soret R (2010). Short-chain fatty acids regulate the enteric neurons and control gastrointestinal motility in rats. Gastroenterology.

[19] Sun M, Wu W, Liu Z, Cong Y (2017). Microbiota metabolite short chain fatty acids, GPCR, and inflammatory bowel diseases. J. Gastroenterol..

[20] Scheppach W (1994). Effects of short chain fatty acids on gut morphology and function. Gut.

[21] McNabney S, Henagan T (2017). Short chain fatty acids in the colon and peripheral tissues: a focus on butyrate, colon cancer, obesity and insulin resistance. Nutrients.

[22] Cani PD (2007). Metabolic endotoxemia initiates obesity and insulin resistance. Diabetes.

[23] Lin HV (2012). Butyrate and propionate protect against diet-induced obesity and regulate gut hormones via free fatty acid receptor 3-independent mechanisms. PloS one.

[24] Gao, Z. *et al*. Butyrate improves insulin sensitivity and increases energy expenditure in mice. *Diabetes* (2009). 10.2337/db08-1637 PMC2699871 19366864

[25] Khan S, Jena G (2014). Protective role of sodium butyrate, a HDAC inhibitor on beta-cell proliferation, function and glucose homeostasis through modulation of p38/ERK MAPK and apoptotic pathways: study in juvenile diabetic rat. Chem-Biol. Interact..

[26] Jakobsdottir G, Xu J, Molin G, Ahrne S, Nyman M (2013). High-fat diet reduces the formation of butyrate, but increases succinate, inflammation, liver fat and cholesterol in rats, while dietary fibre counteracts these effects. PloS one.

[27] Yadav H, Lee J-H, Lloyd J, Walter P, Rane SG (2013). Beneficial metabolic effects of a probiotic via butyrate induced GLP-1 secretion. J. Biol. Chem., jbc..

[28] de Goffau MC (2014). Aberrant gut microbiota composition at the onset of type 1 diabetes in young children. Diabetologia.

[29] Endesfelder D (2016). Towards a functional hypothesis relating anti-islet cell autoimmunity to the dietary impact on microbial communities and butyrate production. Microbiome.

[30] Kluytmans J, Van Belkum A, Verbrugh H (1997). Nasal carriage of Staphylococcus aureus: epidemiology, underlying mechanisms, and associated risks. Clin. Microbiol. Rev..

[31] Round, J. L. *et al*. The Toll-like receptor 2 pathway establishes colonization by a commensal of the human microbiota. *Science*, 1206095 (2011). 10.1126/science.1206095 PMC3164325 21512004

[32] Furman BL (2015). Streptozotocin‐induced diabetic models in mice and rats. Curr. Protoc. Pharmacol..

[33] King AJ (2012). The use of animal models in diabetes research. British journal of pharmacology.

[34] Somboonwong J, Traisaeng S, Saguanrungsirikul S (2015). Moderate-intensity exercise training elevates serum and pancreatic zinc levels and pancreatic ZnT8 expression in streptozotocin-induced diabetic rats. Life Sci..

[35] Akiba Y (2017). FFA2 activation combined with ulcerogenic COX inhibition induces duodenal mucosal injury via the 5-HT pathway in rats. Am. J. Physiol-Gastrl..

[36] Kim JE (2012). Enhancing acid tolerance of Leuconostoc mesenteroides with glutathione. Biotechnol. Lett..

[37] Johanningsmeier S, McFeeters RF, Fleming HP, Thompson RL (2007). Effects of Leuconostoc mesenteroides starter culture on fermentation of cabbage with reduced salt concentrations. J. Food Sci..

[38] Allameh SK, Daud H, Yusoff FM, Saad CR, Ideris A (2012). Isolation, identification and characterization of Leuconostoc mesenteroides as a new probiotic from intestine of snakehead fish (Channa striatus). Afr. J. Biotechnol..

[39] Seo B (2012). Evaluation of Leuconostoc mesenteroides YML003 as a probiotic against low-pathogenic avian influenza (H9N2) virus in chickens. J. Appl. Microbiol..

[40] Kekkonen RA (2008). Probiotic Leuconostoc mesenteroides ssp. cremoris and Streptococcus thermophilus induce IL-12 and IFN-γ production. World J. Gastroenterol..

[41] Li X (2017). Effects of Lactobacillus casei CCFM419 on insulin resistance and gut microbiota in type 2 diabetic mice. Benef. Microbes.

[42] Li C (2014). Carrot juice fermented with Lactobacillus plantarum NCU116 ameliorates type 2 diabetes in rats. J. Agr. Food Chem..

[43] Chen P (2014). Oral administration of Lactobacillus rhamnosus CCFM0528 improves glucose tolerance and cytokine secretion in high-fat-fed, streptozotocin-induced type 2 diabetic mice. J. Funct. Foods..

[44] Kozakova H, Schwarzer M, Srutkova D, Hudcovic T, Cukrowska B (2014). Colonisation of germ-free mice with probiotic lactobacilli mitigated allergic sensitisation in murine model of birch pollen allergy. Clinical and translational allergy.

[45] Zarrinpar A (2018). Antibiotic-induced microbiome depletion alters metabolic homeostasis by affecting gut signaling and colonic metabolism. Nature communications.

[46] Regard JB (2007). Probing cell type–specific functions of G i *in vivo* identifies GPCR regulators of insulin secretion. J. Clin. Invest..

[47] Ahren B (2009). Islet G protein-coupled receptors as potential targets for treatment of type 2 diabetes. Nat. Ref. Drug Discov..

[48] Leonard, J. N. & Hakak, Y. (Google Patents, 2010).

[49] Layden BT (2010). Regulation of pancreatic islet gene expression in mouse islets by pregnancy. J. Endocrinol..

[50] Ulven T (2012). Short-chain free fatty acid receptors FFA2/GPR43 and FFA3/GPR41 as new potential therapeutic targets. Front. Endocrinol..

[51] Fuller M (2015). The short-chain fatty acid receptor, FFA2, contributes to gestational glucose homeostasis. Am. J. Physiol-Endoc. M..

[52] McNelis, J. C. *et al*. GPR43 potentiates beta cell function in obesity. Diabetes, db141938 (2015). 10.2337/db14-1938 PMC4542437 26023106

[53] Duckworth WC, Bennett RG, Hamel FG (1998). Insulin degradation: progress and potential. Endocrine reviews.

[54] Jin CJ, Sellmann C, Engstler AJ, Ziegenhardt D, Bergheim I (2015). Supplementation of sodium butyrate protects mice from the development of non-alcoholic steatohepatitis (NASH). British Journal of Nutrition.

[55] Raso, G. M. *et al*. Effects of sodium butyrate and its synthetic amide derivative on liver inflammation and glucose tolerance in an animal model of steatosis induced by high fat diet. *PloS one* **8** (2013). 10.1371/journal.pone.0068626 PMC3702592 23861927

[56] Matis G (2015). Effects of oral butyrate application on insulin signaling in various tissues of chickens. Domestic animal endocrinology.

[57] Boros LG, Collins TQ, Somlyai G (2017). What to eat or what not to eat—that is still the question. Neuro-oncology.

[58] Davie JR (2003). Inhibition of histone deacetylase activity by butyrate. The Journal of nutrition.

[59] Mariño E (2017). Gut microbial metabolites limit the frequency of autoimmune T cells and protect against type 1 diabetes. Nat. Immunol..

[60] Bindels LB, Dewulf EM, Delzenne NM (2013). GPR43/FFA2: physiopathological relevance and therapeutic prospects. Trends Pharmacol. Sci..

[61] Han J (2008). Extracellular high-mobility group box 1 acts as an innate immune mediator to enhance autoimmune progression and diabetes onset in NOD mice. Diabetes.

[62] Zhang S, Zhong J, Yang P, Gong F, Wang C-Y (2010). HMGB1, an innate alarmin, in the pathogenesis of type 1 diabetes. Int. J. Clin. Exp. Patho..

[63] Abdel-Moneim A, Bakery HH, Allam G (2018). The potential pathogenic role of IL-17/Th17 cells in both type 1 and type 2 diabetes mellitus. Biomed. Pharmacother..

[64] Oh YS, Lee YJ, Park EY, Jun HS (2011). Interleukin-6 treatment induces beta‐cell apoptosis via STAT-3-mediated nitric oxide production. Diabetes Metab. Res. Rev..

[65] Guo Y (2018). Sodium butyrate ameliorates streptozotocin-induced type 1 diabetes in mice by inhibiting the HMGB1 expression. Front. Endocrinol..

[66] Hansen AH (2017). Development and characterization of a fluorescent tracer for the free fatty acid receptor 2 (FFA2/GPR43). J. Med. Chem..

[67] Eleazu CO, Eleazu KC, Chukwuma S, Essien UN (2013). Review of the mechanism of cell death resulting from streptozotocin challenge in experimental animals, its practical use and potential risk to humans. J. Diabetes Metab. Disord..

[68] Pingitore A (2019). Short chain fatty acids stimulate insulin secretion and reduce apoptosis in mouse and human islets *in vitro*: Role of free fatty acid receptor 2. Diabetes Obes. Metab..

[69] Pace BS (2002). Short-chain fatty acid derivatives induce fetal globin expression and erythropoiesis *in vivo*. Blood.

[70] Matheus V, Monteiro L, Oliveira R, Maschio D, Collares-Buzato C (2017). Butyrate reduces high-fat diet-induced metabolic alterations, hepatic steatosis and pancreatic beta cell and intestinal barrier dysfunctions in prediabetic mice. Experimental Biology and Medicine.

