# Supplementary material for: Leuconostoc mesenteroides fermentation produces butyric acid and mediates Ffar2 to regulate blood glucose and insulin in type 1 diabetic mice
Source: Sci Rep. 2020 May 13;10:7928. doi: 10.1038/s41598-020-64916-2 (PMC7220903; doi:10.1038/s41598-020-64916-2)
Supplement: Supplementary file 1 — Supplementary Information. [file 41598_2020_64916_MOESM1_ESM.docx]

***Leuconostoc mesenteroides* fermentation produces butyric acid and mediates Ffar2 to regulate blood glucose and insulin in type 1 diabetic mice**

Supitchaya Traisaeng^1^, Anir Batsukh^2^, Tsung-Hsien Chuang^3,^ Deron Raymond Herr^4^, Yu-Fen Huang^5^, Battogtokh Chimeddorj^6^, and Chun-Ming Huang^2*^

^1^ Department of Life Sciences, National Central University, Taoyuan, Taiwan

^2^ Department of Biomedical Sciences and Engineering, National Central University, Taoyuan, Taiwan

^3^ Immunology Research Center, National Health Research Institutes, Miaoli, Taiwan

^4^ Department of Pharmacology, National University of Singapore, Singapore

^5^ Department of Biomedical Engineering and Environmental Sciences, National Tsing Hua University, Taiwan

^6^ Department of Microbiology and Immunology, Mongolian National University of Medical Sciences, Mongolia

***Corresponding author:** Professor Chun-Ming Huang, Ph.D.

Department of Biomedical Sciences and Engineering, National Central University, Taoyuan; Tel: +886-3-422-7151 x 36101; Fax: +886-3-425-3427; Email: chunming@ncu.edu.tw

**Methods**

**HPLC analysis.** Cultured media or mouse cecum homogenates were centrifuged at 5,000 rpm for 10 min. The supernatants were filtered through a 0.22 μm microfiltration membrane to remove bacteria and all insoluble particles. The filtrates were vortexed and equilibrated at room temperature for 5 min. Thereafter 100 μL of concentrated HCl was added, followed by a vortex mixing step of 15 s. The samples were extracted for 20 min (by gently rolling) using 5 mL of diethyl ether. After centrifugation (5 min, 3,500 rpm), the supernatant was transferred to another pyrex extraction tube before 500 μL of a 1 mol/L solution of NaOH was added. The samples were extracted again for 20 min, followed by a centrifugation step. The aqueous phase was transferred to an autosampler vial and 100 μL of concentrated HCl were added. The analysis of butyric acid was performed using an Agilent 1200 series HPLC system with a ZORBAX Eclipse XDB-C18 column (4.6 × 250 mm, 5 μm). The temperature of the column oven was set at 30°C. The mobile phase consisted of 20 mmol/L NaH_2_PO_4_ solution (pH 2.2) and acetonitrile. The flow rates were 0.3 and 0.5 mL/min for samples of culture media and mouse cecum, respectively. The detector was set at 210 nm. The injection volume was 50 μL. The concentrations of butyric acid were calculated according to calibration curves of a butyric acid analytical standard.

**Immunohistochemical analysis of pancreatic insulin content**. Pancreas sections were deparaﬃnized with xylene and ethanol for 10 min. After water washing, antigen retrieval was performed in citrate buffer with ethylenediaminetetraacetic acid (EDTA) for 30 min using a steamer target retrieval solution in a microwave oven. Next, the slides were incubated with 3% hydrogen peroxide for 5 min to inhibit endogenous peroxidase activity, followed by 3% normal goat serum for 20 min to block nonspeciﬁc binding. After that, the primary antibody specific to insulin (Abcam, Cambridge, MA, USA; diluted 1:16,000) was applied, and incubated with horseradish peroxidase-conjugated goat anti-rabbit IgG (Abcam, Cambridge, MA, USA; diluted 1:500) and then with 3,3-diaminobenzidine. The slides were counterstained with hematoxylin. Under light microscopy, the positively stained cells presented dark brown cytoplasm. The results were expressed as the number of positively stained cells per ﬁeld. The specificity of insulin immunoreactivity was confirmed by omitting the primary antibodies from some sections. Images were analyzed using Image J software (National Institutes of Health, Bethesda, Maryland, USA) by measuring the average pixel value of staining per cell and per islet and the value obtained was deduced by the background staining value. Approximately 5 islets per section were randomly selected from each mouse. The method of measuring OD for immunoreactive insulin content was modified from those previously described by previous studies ^1^.

**Effects of temperatures and pH values on the growth of *L. mesenteroides* EH-1.** *L. mesenteroides* EH-1 (10^7^ CFU/mL) was cultured in TSB at 4°C, 25°C or 37°C. The growth of *L. mesenteroides* EH-1 was measured by an absorbance measurement (OD_600_ nm) 0, 12, 24, 36, 48, and 60 h after culture. *L. mesenteroides* EH-1 (10^7^ CFU/mL) was incubated in TSB at pH 2-7 for 90 min. After incubation, bacteria were counted by plating serial dilutions (1∶10^0^ to 1∶10^5^) of the cultured media on a plate. The number of bacteria was quantified by spotting the dilution (10 μL) on an agar plate supplemented with media for the counting of CFUs.

**OGTT.** STZ-induce diabetic ICR mice were injected with butyric acid (5 mL/kg body weight) or water. Butyric acid was taken from a stock solution at the concentration of 4 mmol/L. After 24 h of first injection, the second injection (butyric acid or water) was performed right before oral administration of glucose (1.5 g/kg body) for OGTT. Mice were fasted for 6 h before oral administration of glucose. The tail blood of mice was drawn and glucose was measured at 0, 15, 30, 60, and 120 min after administration of glucose^2^. After 120 min, the orbital sinus blood was collected for measurement of insulin by ELISA.

**RT-PCR.** RNA (100 ng) extracted from Min6 cells was converted into cDNA using an iScript cDNA Synthesis Kit (Bio-Rad, Hercules, CA, USA). All sets were designed using National Center for Biotechnology Information (NCBI) Primer-Blast (<https://www.ncbi.nlm.nih.gov/tools/primerblast/>). The reaction was performed on StepOnePlus Real-Time PCR System (Thermo Fisher Scientific) using Power SYBRGreen PCR Master Mix (Thermo Fisher Scientific). The reaction conditions for 40 cycles are as follows: 95°C for 10 min followed by 95°C for 15 s, 50°C for 60 s, and 72°C for 30 s. Gene expression of glyceraldehyde 3-phosphate dehydrogenase (GAPDH) was used for normalization. The relative expression levels were calculated using the delta delta cycle threshold (∆∆Ct). Primers used for GAPDH and Ffar2 were 5’-TGTGTCCGTCGTGGATCTGA-3’ (forward); 5’-GATGCCTGCTTCACCACCTT-3’ (reverse) and 5’-ACCCAAGAGCAGCTGGATGT-3’ (forward); 5’-AGCGCCAATAACAGAAGATGGT-3’ (reverse), respectively.

**References**

1 Huang, H.-H. *et al.* Exercise increases insulin content and basal secretion in pancreatic islets in type 1 diabetic mice. *Eep. Diabetes Res.* **2011** (2011).

2 Wei, S.-H., Chen, Y.-P. & Chen, M.-J. Selecting probiotics with the abilities of enhancing GLP-1 to mitigate the progression of type 1 diabetes in vitro and in vivo. *J. Funct. Foods.* **18**, 473-486 (2015).

**Legends of supplementary Table and figures**

**Supplementary Table 1.** The mean values ± SD for all figures with bar charts.

**Supplementary Fig. S1.** The nucleotide sequences of 16S rRNA gene of *L. mesenteroides* EH-1 isolated from Mongolian curd cheese.

**Supplementary Fig. S2.** The growth of *L. mesenteroides* EH-1 at different temperatures. *L. mesenteroides* EH-1 was cultured at 4°C (brown), 25°C (gray) or 37°C (black) for 60 h. Data are the mean ± SD from 3 separate experiments. **p* < 0.05; ***p* < 0.01; *** *p* < 0.001 vs 37 °C and **^###^***p* < 0.001 vs 4°C

**Supplementary Fig. S3.** The effect of pH values on *L. mesenteroides* EH-1 growth. *L. mesenteroides* EH-1 (10^7^ CFU/mL) was incubated in TSB at pH 3-7 for 90 min. (**a**) After incubation, *L. mesenteroides* EH-1 was counted by plating serial dilutions (1∶10^0^ to 1∶10^5^) of the cultured media on an agar plate. (**b**) The number (log_10_ CFU/mL) of *L. mesenteroides* EH-1*.* The CFU counts were illustrated as mean ± SD from experiments in triplicate.

**Supplementary Fig. S4.** Change in the level of glucose, insulin and IL-6 in diabetic mice. The levels of fasting blood glucose (**a**), insulin (**b**) and IL-6 (**c**) in mice injected without (C, white, n = 4) or with STZ (DM, black, n = 4). Data are the mean ± SD from 3 independent experiments. ****p* < 0.001

**Supplementary Fig. S5.** The expression of Ffar2 in Min6 cells pre-treated with Ffar2 or negative control siRNAs. The gene expression of the Ffar2 relative to the GAPDH in Min6 cells with/without siRNA knockdown was quantified by RT-PCR. Data are the mean ± SD obtained for three independent experiments. ****p* < 0.001

**Supplementary Fig. S6.** Effects of propionic and butyric acids on insulin secretion from Min6 cells. (**a**) Levels of insulin secretion from Min6 cells after treatments with water (H_2_O), 100 µmol/L propionic acid (PA), or 100 µmol/L butyric acid (BA) for 24 h were measured by ELISA. (**b)** Min6 cells pre-treated with Ffar2 or negative control siRNAs before incubation with water, propionic acid, or butyric acid for 24 h. The level (µg/L) of insulin was detected using a mouse insulin ELISA kit. Data present the mean ± SD for 3 independent experiments. ****p* < 0.001 vs water treatment and ## *p* < 0.01; ###*p* < 0.001 vs propionic acid treatment

**Supplementary Fig. S7.** No effect of killed *L. mesenteroides* EH-1 on the levels of blood glucose and insulin. Diabetic mice were fed with killed *L. mesenteroides* EH-1 (killed-LM; solid square/bar or water (H_2_O; open square/bar) once a day for 2 weeks. The levels of (**a**) glucose were detected once a week, and (**d**) insulin was measured after 2 weeks. Data are the mean ± SD for three independent experiments with four mice per group.

**Supplementary Fig. S8.** Detection of glucose metabolism and insulin level in mice by OGTT. Diabetic mice were injected with water (H_2_O; open square/bar) or butyric acid (BA; solid square/bar) for 24 h before oral administration of glucose. (**a**) Blood glucose was quantified at 0, 15, 30, 60 and 120 min after glucose administration. After 120 min, (**b**) Blood was collected for insulin detection by ELISA. 120 min after glucose administration. Data are the mean ± SD from three separate experiments with 3 mice per group. **p* < 0.05; ***p* < 0.01

**Supplementary Table and figures**

**Supplementary Table 1.**

**
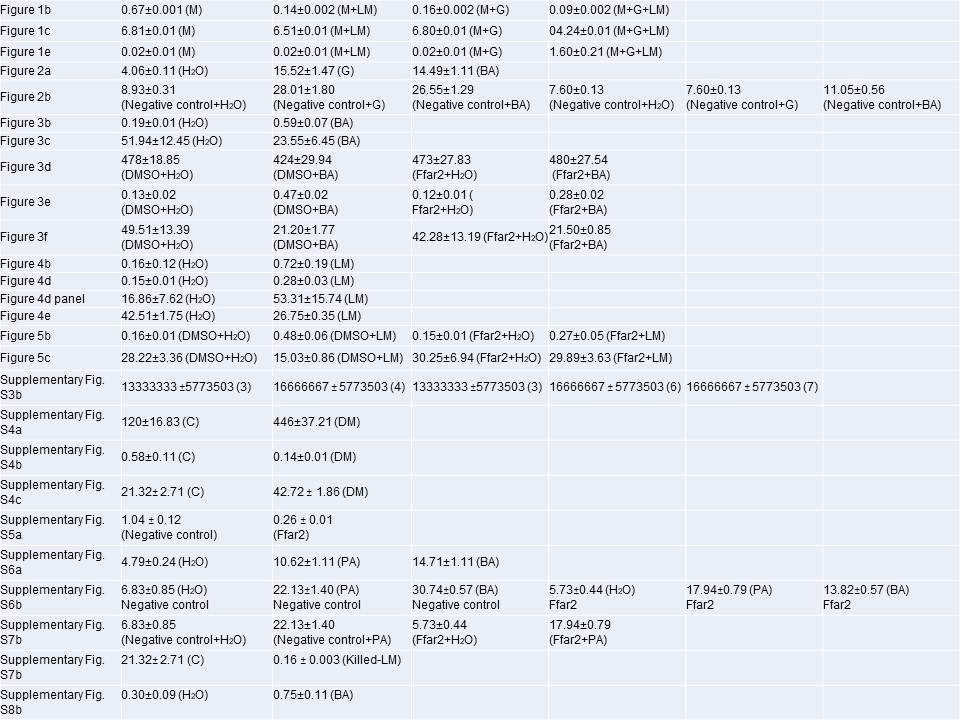
**

**Supplementary Fig. S1.**


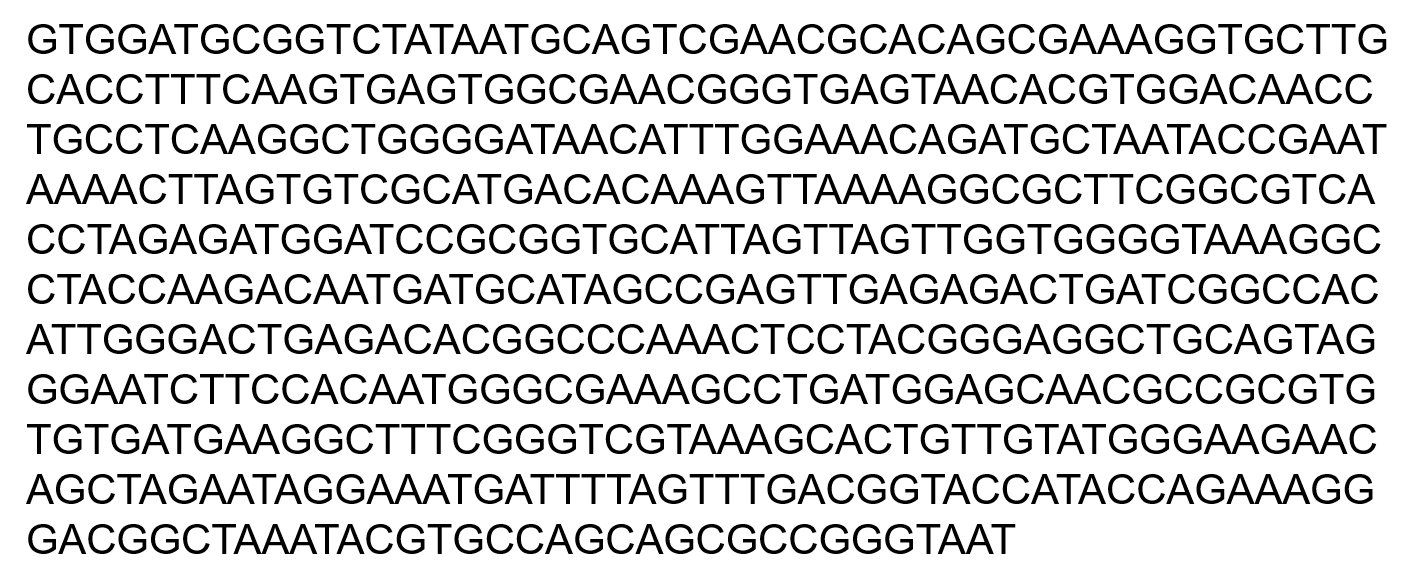


**Supplementary Fig. S2.**

**
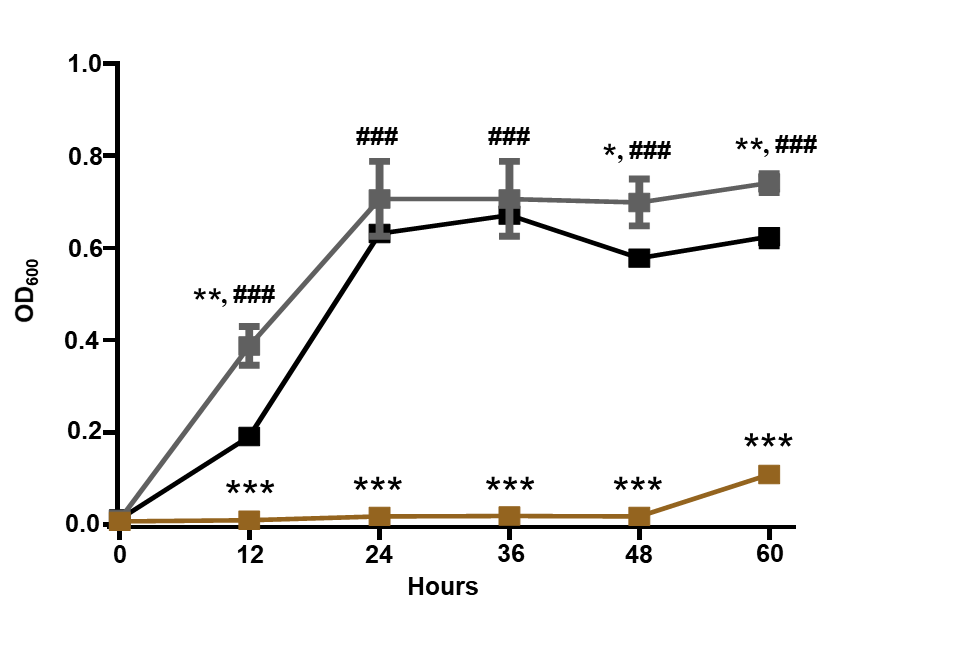
**

**Supplementary Fig. S3.**


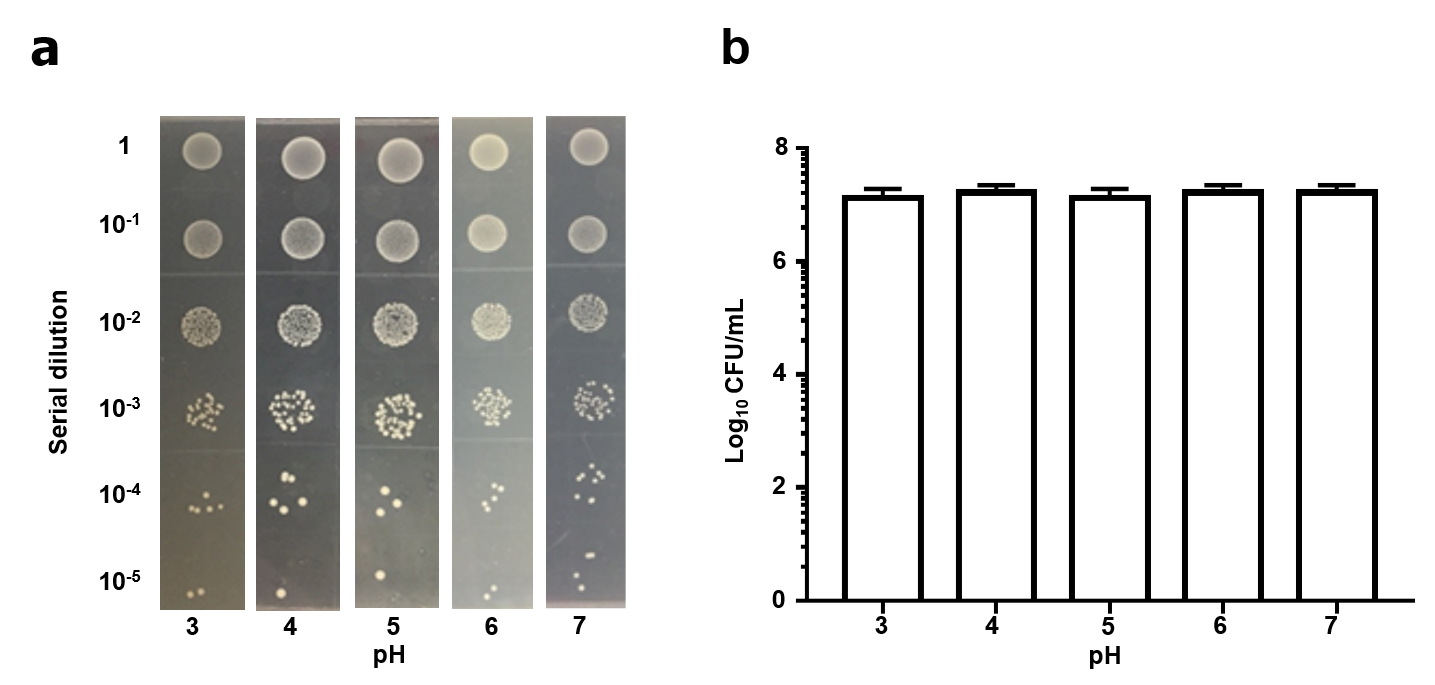


**Supplementary Fig. S4.**

**
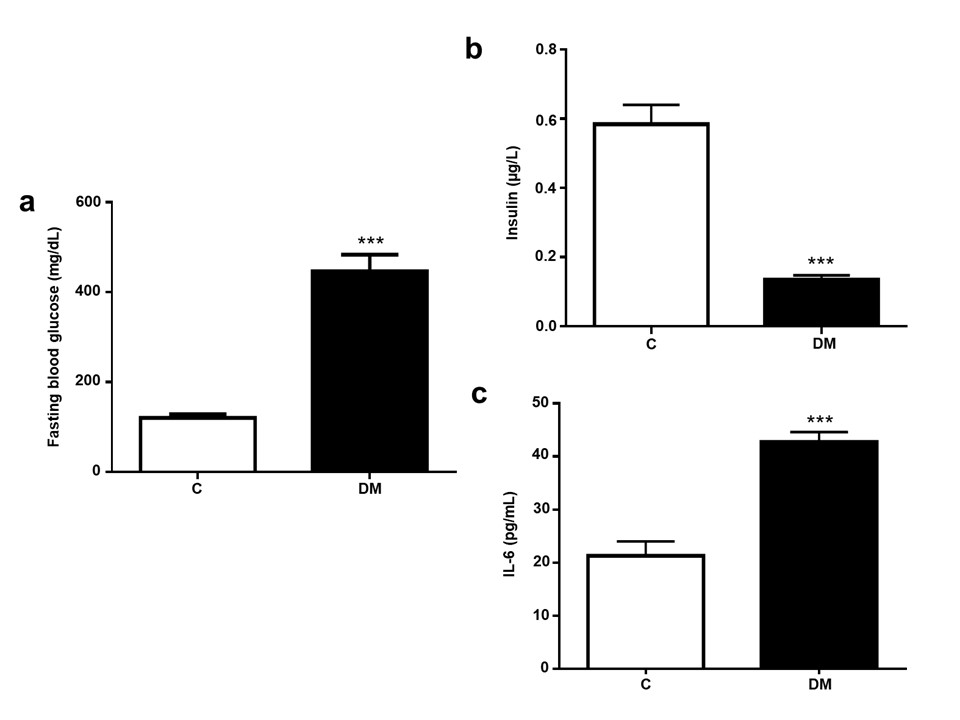
**

**Supplementary Fig. S5.**

**
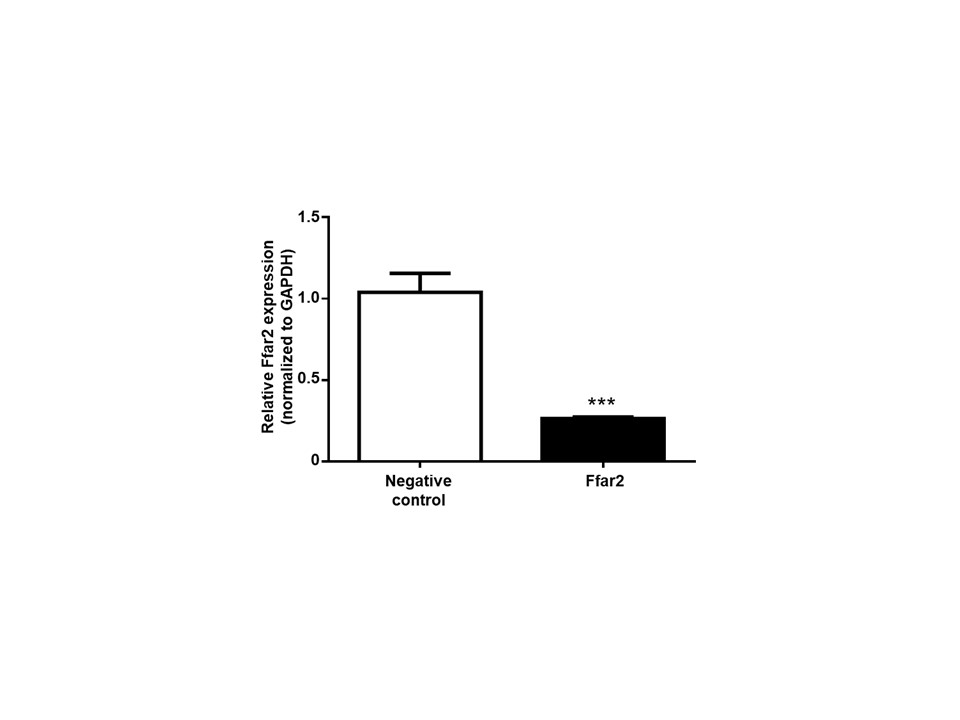
**

**Supplementary Fig. S6.**


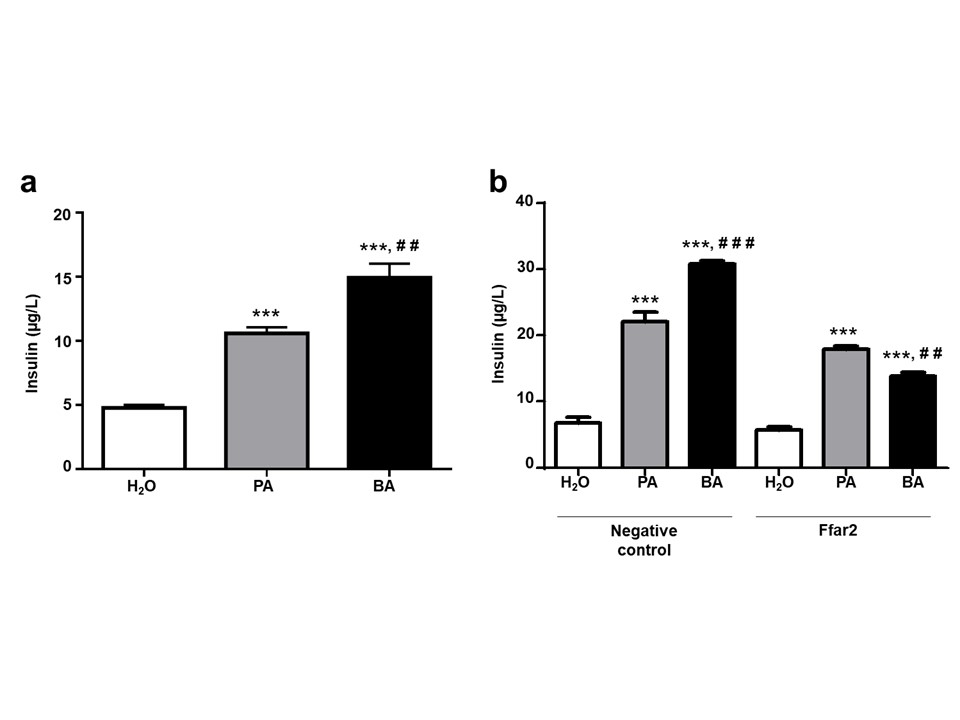


**Supplementary Fig. S7.**

**
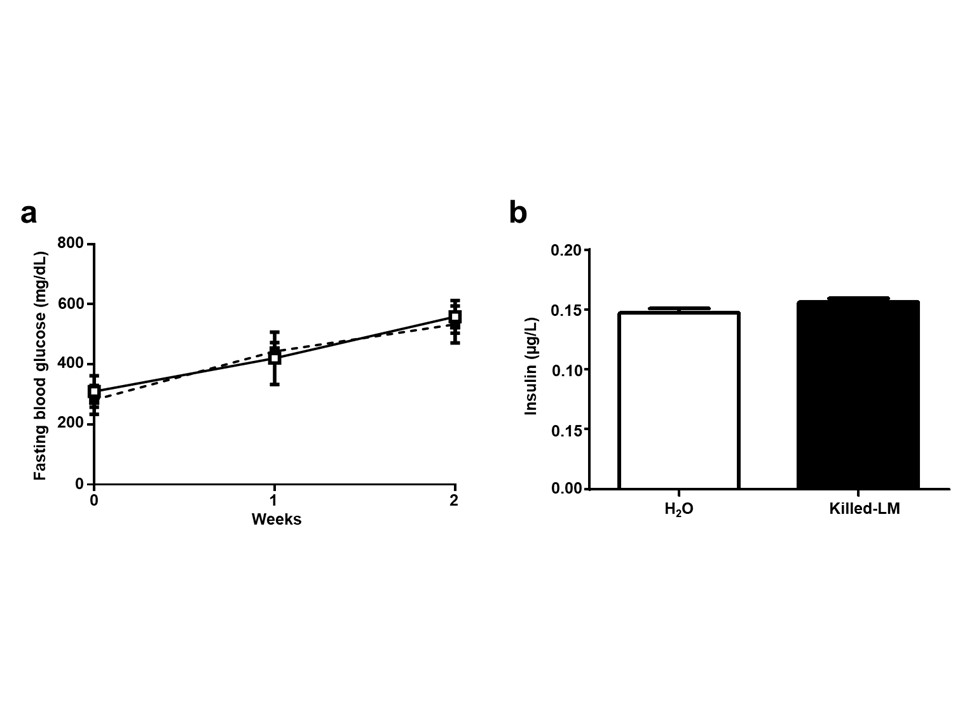
**

**Supplementary Fig. S8.**

**
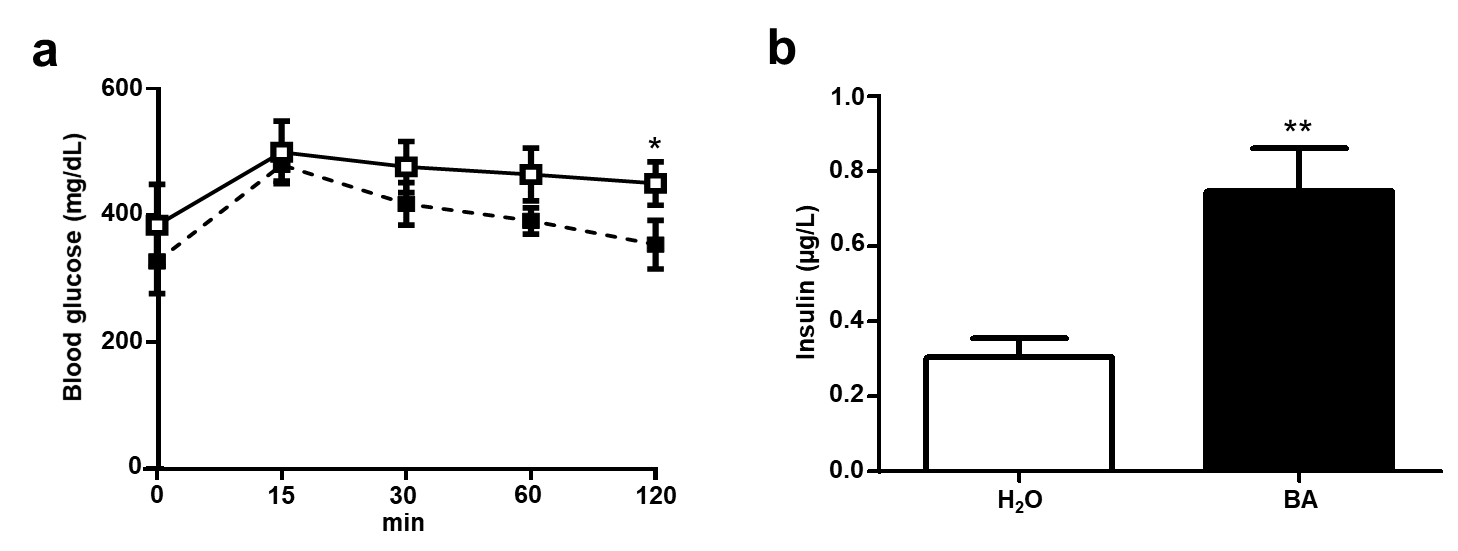
**
